# Microbial warfare and the evolution of symbiosis

**DOI:** 10.1098/rsbl.2022.0447

**Published:** 2022-12-21

**Authors:** Matishalin Patel, Stuart West

**Affiliations:** ^1^ Centre for the Future of Intelligence, University of Cambridge, Cambridge, Cambridgeshire CB2 1SB, UK; ^2^ Department of Zoology, University of Oxford, Oxford OX1 2JD, UK

**Keywords:** microbial, evolution, symbiosis, conflict, mutualisms

## Abstract

Cooperative symbionts enable their hosts to exploit a diversity of environments. A low genetic diversity (high relatedness) between the symbionts within a host is thought to favour cooperation by reducing conflict within the host. However, hosts will not be favoured to transmit their symbionts (or commensals) in costly ways that increase relatedness, unless this also provides an immediate fitness benefit to the host. We suggest that conditionally expressed costly competitive traits, such as antimicrobial warfare with bacteriocins, could provide a relatively universal reason for why hosts would gain an immediate benefit from increasing the relatedness between symbionts. We theoretically test this hypothesis with a simple illustrative model that examines whether hosts should manipulate relatedness, and an individual-based simulation, where host control evolves in a structured population. We find that hosts can be favoured to manipulate relatedness, to reduce conflict between commensals via this immediate reduction in warfare. Furthermore, this manipulation evolves to extremes of high or low vertical transmission and only in a narrow range is partly vertical transmission stable.

## 1. Introduction

Symbiotic cooperation between species allows new niches to be exploited and the evolution of more complex life [1,2]. Aphids can grow feeding only on nutrient-poor plant sap, because their symbiotic bacteria provide essential amino acids [3]. The siboglinid worms depend upon methane-oxidizing and sulfide-eating symbionts to survive near hydrothermal vents [4]. Symbiosis between an archaeal host cell and an aerobic bacterium gave rise to the eukaryotes [1,5].

Both theory and empirical data have suggested that a high genetic relatedness between the symbionts within a host can play a key role in promoting symbiotic cooperation. When symbionts are more closely related, any benefits received from increasing host growth are more likely to be shared with relatives instead of unrelated non-producers, which increases the kin-selected benefit of providing help to hosts [6–10]. Consistent with this prediction, across different symbioses, symbionts appear to provide more help to their host when relatedness is higher [11]. Additionally, experimental and observational studies have shown that conditions which lead to a higher relatedness also lead to greater cooperation [12–14].

However, Frank [15] pointed out that although a higher relatedness would favour symbionts (or commensals) to become more cooperative, hosts would not necessarily be selected to maintain associations with their symbionts in ways that increased relatedness. The reason for this is that, assuming cooperation was not adjusted conditionally in response to the local relatedness, symbionts would only evolve a higher level of cooperation in response to relatedness over time. Consequently, all else being equal, there would be no immediate fitness benefit to investing in costly vertical transmission of symbionts that increased relatedness [16,17]. So, hosts that invested in costly vertical transmission will be less fit than those that do not, and the mutation would not spread.

We suggest that competition through bacteriocins could provide a relatively general explanation why hosts would be selected to increase the relatedness between bacterial symbionts. Empirical work has shown that some bacterial species conditionally upregulate their production of bacteriocins in response to the presence of competing strains [18–20]. Consequently, when there are more genetically differentiated lineages, and so relatedness between interacting bacterial symbionts is lower, bacterial symbionts will put more resources into killing each other. This increased bacteriocin production will reduce growth, such that the population of symbionts will be less beneficial to their hosts [21]. We suggest that hosts will be selected to transmit or house their symbionts in ways that increased relatedness, to decrease this costly conflict [22]. Our argument focuses on bacteriocins as an example, but this mechanism could more generally apply whenever the symbiont species express competitive traits that are costly, and which are conditionally modulated. We theoretically model the plausibility of this hypothesis and determine the conditions under which it would be most favoured. We find that hosts can evolve costly control and that this control can evolve to high levels, leading to a high relatedness between symbionts.

## 2. Model

### (a) Analytical model

We first developed a deliberately simple analytical model to illustrate the general features of our proposed mechanism. Empirical data have shown that when bacteria encounter competing strains, they increase their production of bacteriocins [18–20]. Therefore, when more bacterial strains infect a host the growth rate of each strain will be reduced for two reasons: (i) they will be investing more resources into bacteriocin production and (ii) the mortality caused by bacteriocins from other strains will be greater. We capture these effects by assuming that symbiont growth is a positive function of the relatedness within the group:

2.1 $$\begin{array}{c} S(r)\propto r^{\alpha }. \end{array}$$

The parameter *α* determines whether the relationship is linear (*α* = 1), decelerating (0 < *α* < 1) or accelerating (*α* > 1). This shape parameter captures all the details of how the production and effect of the bacteriocin interact to influence the growth rate of a symbiont.

We assume in the model that hosts gain more benefit from their bacterial symbionts when these symbionts are better able to grow. Hosts invest in a costly manipulation to influence relatedness among their symbionts. This manipulative trait (g∈[0,1]) could be a behaviour, such as young eating parental faeces, or a structural adaptation, such as specialized organs in the host to store and transmit symbionts [23].

The initial relatedness among symbionts infecting a host is *r_s_* ∈ [0, 1]. This starting relatedness could be due to existing behaviours, structural constraints or transmission routes. We assume that if a host invests an amount *g* ∈ [0, 1] in a costly manipulation trait that this increases relatedness to some final relatedness (*r_f_* ∈ [0, 1]):

2.2 $$\begin{array}{c} r_{f}=r_{s}+g(1-r_{s}). \end{array}$$

We assume that the cost of investing in the trait that increases symbiont relatedness reduces host fitness by a fraction (1 − *g*)*^β^*, where *β* is a shape parameter (*β* = 1, linear; 0 < *β* < 1, decelerating; *β* > 1, accelerating). Our model focuses on relatedness and makes no assumptions about the relative amounts of vertical or horizontal transmission. For example, all transmission could be vertical, with the costly manipulation trait altering the diversity of transmitted symbionts.

We assume that the cost of increasing symbiont relatedness and benefits from symbiont growth interact multiplicatively, such that host fitness (*W_H_*) is

2.3 $$\begin{array}{c} W_{H}={\left(1-g\right)}^{\beta }\;{\left(r_{s}+g(1-r_{s})\right)}^{\alpha }. \end{array}$$

We aim to find when this costly trait is favoured. To do this, we solve for a candidate evolutionarily stable strategy (ESS) amount of manipulation for the host to invest in (g*). The ESS (g*) represents the strategy which could not be beaten by any other strategy and is given by solving $dW_{H}/dg\|{\;}_{g=g^{*}}=0$ [24], to find the stationary points and $d^{2}W_{H}^{2}/dg^{2}\|{\;}_{g=g^{*}}\leq 0$, to confirm it is a maximum (see appendix). This approach does not make assumptions about sexual system or ploidy of the host or symbiont [25]. We found that

2.4 $$\begin{array}{c} g^{*}=1-\frac{\beta }{\left(\alpha +\beta )(1-r_{s}\right)}. \end{array}$$

Equation (2.4) predicts that hosts can be favoured to perform a costly trait that increases relatedness between symbionts, in order to reduce symbiont investment in costly conflict. Specifically, the ESS host manipulation of relatedness (g*) will increase when: (i) the starting relatedness among symbionts decreases (*r_s_*), (ii) the influence of relatedness on symbiont growth is more accelerating (higher *α*) and (iii) the cost of manipulating relatedness is more decelerating (lower *β*).

### (b) Simulations

We then developed an individual-based simulation model to relax some of the assumptions of our analytical model. We assumed a set of patches, which each contain a fixed number of asexual hosts and free-living bacteria. Each host is colonized by a fixed number of bacteria. The host population and the bacterial population each time step underwent a birth–death/death–birth process with the probability of reproducing being proportional to fitness (death was always random). The bacterial population experienced five time steps for every one time step of the host to simulate a faster life cycle, for nearly neutral mutations this is approximately equal to bacteria having a fivefold increased mutation rate. Each host could invest in a control trait *g* ∈ [0, 1]. Every time a host reproduced the symbionts for its child were drawn one at a time randomly. With probability Λ = *λ* + *g*(1 − *λ*), a new symbiont was drawn from the mother and with probability 1 − Λ, a new symbiont was drawn from the free-living symbiont population; *λ* is the basic vertical transmission chance due to environmental and biological factors and did not change during a single simulation run. In this simulation, we therefore consider the special case where host control arises by altering the relative probability of vertical and horizontal transmission. Additionally, there was a shedding term (*σ*) each time step that reintroduced host bacteria into the free-living population with every time step. All sampling was weighted by fitness relative to their respective populations (either free-living or the mother's symbiont population).

The symbiont fitness was calculated using the following expression:

2.5 $$\begin{array}{c} W_{S}=(1-\bar{x})\frac{x}{\bar{x}\;}. \end{array}$$

Where *x* is the symbiont's bacteriocin production, and $\bar{x}$ is the group average bacteriocin production. This expression has a well-known ESS of x* = 1 − *R* (appendix A) [7]. We assume bacteria have a conditional rule (*x_C_*) that uses this ESS strategy where they play the strategy *x_C_* = (1 − *R*), where *R* is directly measured using strain identity tags that are tracked in the simulation (*x_C_* was clamped to the range [1 × 10^−6^, 1 − 1 × 10^−6^] to prevent division by 0).

Host fitness was derived from symbiont bacteriocin production:

2.6 $$\begin{array}{c} W_{H}=(1-\bar{x})(1-cg). \end{array}$$

Where $\bar{x}$ is the average bacteriocin produced by the symbionts within the host, *g* is the host control trait and *c* is the cost per unit of investment in control (*g*).

**Figure 1.**
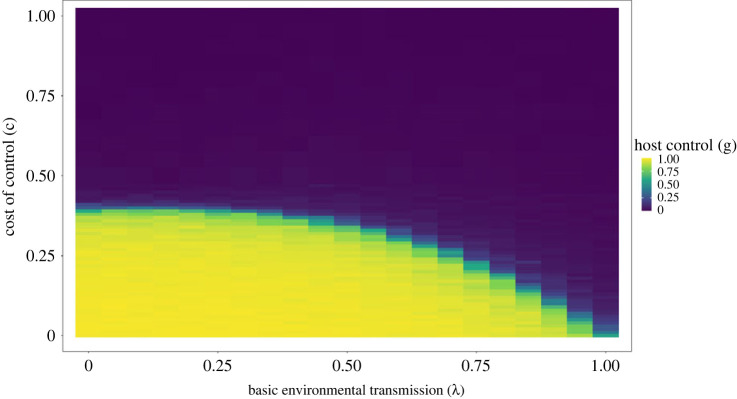
Host control (yellow region) evolves when the cost of control is low and when basic environmental transmission is low. When basic transmission is high additional control is not selected for and when the cost is too high control is also disfavoured. The region transitions sharply between high control (*g* > 0.9) and low control (*g* < 0.1) with intermediate control only being favoured in a narrow border region between the two zones.

**Figure 2.**
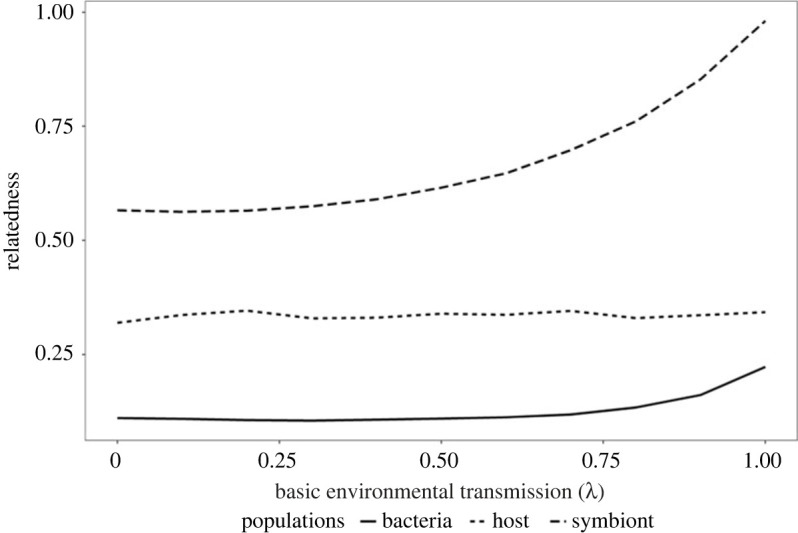
The effect of basic transmission was calculated with no selection on either symbiont bacteriocin or host control. The average relatedness values for the three populations were calculated. Host relatedness shows no change with increased base vertical transmission which is to be expected. Symbiont relatedness shows a clear accelerating relationship with host control and at high levels of control this also affects free-living bacteria through shedding during reproduction.

In our simulations, we varied the cost of the control trait (*c*) and the basic vertical transmission rate (*λ*). Figure 1 shows how host control levels varied for different basic vertical transmission rates on the *x*-axis and the cost of control on the *y*-axis. Control by the host was favoured when cost was low and basic transmission was also low. At high costs, the trait became too expensive, and at high basic vertical transmission, the benefit to extra control was minimal. The transition between regions where control was favoured or not is sharp with either full control (highly vertical transmission) being favoured or zero control (horizontal transmission). Only in a very narrow range along the boundary were intermediate levels of control stable. This is due to the accelerating relationship between symbiont relatedness and vertical transmission which leads to intermediate strategies being less stable than either zero or full control (figure 2).

## 3. Discussion

Our analytical model and simulation showed that hosts can be favoured to increase the relatedness between the bacteria that they contain, or those they transmit to their offspring. Increasing relatedness provides an immediate fitness benefit to the host because it reduces the extent to which symbionts produce compounds such as bacteriocins and engage in costly conflict with competing symbiont strains. This higher relatedness between symbionts would then favour those symbionts that invest in higher levels of cooperation with their hosts. In addition, our model suggests hosts can be selected to evolve high levels of control, leading to effectively vertical transmission, of a form that can be especially conducive to mutual dependence and major evolutionary transitions (figure 1) [5,26].

Our illustrative analytical model assumed that higher relatedness increases the growth rate of the symbiont. This is based upon experimental work on bacteriocin production in bacteria [17,20]. However, previous theory suggests that bacteriocin production may in fact follow a domed relationship with relatedness [27]. This would mean that increasing relatedness may decrease growth rates depending on the starting relatedness. This implies that in nature the evolution of host control may be constrained by the un-manipulated relatedness as sometimes selection may favour reducing relatedness. Consequently, we might predict that evolution would favour either highly related symbionts or highly unrelated ones to avoid these intermediate relatedness values where conflict is high. The exact predictions could depend upon the mechanisms that bacteria use to detect and attack non-relatives [18–20,28].

Our models predict that hosts will invest more resources into increasing relatedness when the ‘background’ relatedness is lower (low *r_s_*), such as when transmission is naturally horizontal (low *λ*). The diversity of mutualisms in the natural world could allow our predictions to be tested with across species comparative studies. For example, among the mycetocyte symbionts that infect a variety of insects the mode of vertical transmission varies from faecal smearing to specialized structures that transfer the symbionts from the mycetocytes to the ovaries [23]. Previous empirical work on termites and ants has shown that they can be selected to maintain their fungal symbionts at high relatedness, to reduce incompatibility conflicts [16,29]. A comparative approach might aim to evaluate how costly the different transmission behaviours are and what the basic transmission rate without intervention would be. These data could then be used to classify species as either actively shaping their symbionts vertical transmission or incidentally benefiting from a life cycle or environment that allows a naturally high basic transmission rate to exist without intervention. When these host–symbiont associations are maintained by environmental mechanisms or the result of selection on traits not directly relevant to the symbiosis, we would expect low host investment into vertical transmission but still observe symbiotic interactions as the association is maintained through other mechanisms.

The evolution of symbioses appears to be constrained differently in aquatic and terrestrial environments. In aquatic systems, there is a much greater diversity in terms of transmission routes with many obligate symbioses, such as corals using horizontal transmission (though some corals do show vertical transmission) [30,31]. One hypothesis is that aquatic environments favour highly plastic symbiont uptake to allow organisms to adapt to local conditions [32–34]. Another possibility is that symbiont diversity can be favoured to provide a diversity of resources [32,33]. Our model does not include any of these environmental factors which could favour horizontal transmission of highly specialized partners. A key future of extension of our work would be in the inclusion of environmental heterogeneity to test for this evolution of horizontally transferred symbionts.

We have provided theoretical support that conditional expression of bacteriocins and kin discrimination by free-living bacteria may give the initial nudge for the evolution of greater symbiosis between bacteria and their hosts. Our model predicts that host control of relatedness and/or vertical transmission is more likely to evolve when basic relatedness/vertical transmission rates are low. There are several unanswered questions that could be investigated with theoretical, comparative or experimental studies. If the expression of bacteriocins is non-conditional, or follows other theoretical rules, how does that alter the evolution of host control? How does explicitly modelling symbiont benefits change the outcome? To what extent can bacteriocin conflict favour the long-term evolution of mutual dependence which would show the full transition from free-living bacteria to obligate symbionts?

## Data Availability

Code files and csv data are available from the Dryad Digital Repository: https://doi.org/10.5061/dryad.p8cz8w9sv [35].

## Appendix: analytical model

Fitness equation for hosts:

A 1 $$\begin{array}{c} W_{H}={\left(1-g\right)}^{\beta }\;{\left(r_{s}+g(1-r_{s})\right)}^{\alpha }. \end{array}$$

Candidate ESS *g**

A 2 $$\frac{dW_{H}}{dg}|{\;}_{g=g^{*}}=0$$

and

A 3 $$\begin{array}{c} g*=\frac{\beta }{\left(r-1)\;(\alpha +\beta \right)}+1. \end{array}$$

We now evaluate the second derivative with respect to *g* at the candidate ESS to confirm that *g** is a maximum of *W_H_*.

A 4 $$\frac{d^{2}W_{H}}{dg^{2}}{|}_{g=g^{*}}<0,$$

and

A 5 $$\begin{array}{c} 0<-\frac{{\left(r-1\right)}^{2}{\left(\alpha /\alpha +\beta \right)}^{\alpha }{\left(\alpha +\beta \right)}^{3}{\left(-(\beta /(r-1)(\alpha +\beta ))\right)}^{\beta }}{\alpha \;\beta }. \end{array}$$

Which holds true as long as α>0,β>0,0≤r <1.

## Appendix: symbiont behaviour

Fitness equation:

A 6 $$\begin{array}{c} W_{S}=(1-\bar{x})\frac{x}{\bar{x}\;}. \end{array}$$

Using Taylor–Frank method Inclusive fitness becomes:

A 7 $$IF=R\left(-\frac{x(1-\bar{x})}{{\bar{x}}^{2}}-\frac{x}{\bar{x}}\right)+\frac{1-\bar{x}}{\bar{x}}.$$

Then at equilibrium when $\bar{x}=x=x^{*}$.

A 8 $$IF{|}_{\bar{x}=x=x^{*}}=0$$

and

A 9 $$x^{*}=1-R.$$

## Appendix: simulation details

The simulations were carried out in populations of 500. Hosts across 50 patches each with two symbionts and with 20 free-living bacteria per patch. Giving total population of 500 hosts, 1000 symbionts and 1000 free-living bacteria. Each simulation was given 2500 time steps of burn in time, with each time step performing one Moran birth–death/death–birth process per patch hosts and one for free-living bacteria, with a small chance of migration between patches with each birth/death event. So, each time step performed 100 birth–death/death–birth steps total. Total run per simulation was 10 000 time steps and each simulation was repeated five times and the endpoints averaged to create figure 1 and 2. Full code and final CSV file is included in the Dryad repository for this paper [35].

Each host had one evolvable trait (*X*) that controlled the probability that it's offspring would inherit from it rather than sampling from the population at random. In the simulations, symbiont number per host was 2 so with probability *X*^2^ both symbionts would come from the mother and with probability (1 − *X*)^2^ they would both come from the local free-living population, and with probability 2(1 − *X*)*X* one would be horizontal and one vertical.

Symbionts had their toxin production conditionally set using 1 − *R*, where *R* was the local relatedness. This conditional toxin production was then used to update the host and symbiont fitness.

**Table:**

variable	value
number of generations	10 000
time at which hosts evolution begins	2500
starting host trait	0.000001
mutation rate of hosts	0.01
mutation rate of symbionts	0.01
migration rate	0.1
cost of vertical transmission	[0, 0.5] in steps of 0.01,
	(0.5, 1] in steps of 0.05
basis vertical transmission	[0, 1] in steps of 0.05
number of patches	50
number of hosts per patch	10
number of symbionts per host	2
number of free-living bacteria per patch	20
number of bacteria steps per host step	[1, 5]
